# Infraorbital Paresthesia as a Potential Indication for Reconstruction of Pure Blowout Orbital Fractures: A Case Report

**DOI:** 10.1155/crid/9351177

**Published:** 2026-08-12

**Authors:** Mohammad H. Al-Shayyab, Ayham Dahooh, Hazem Alahmad, Motaz Barakat, Sara Abdallah, Lina Ghattas, Majd Y. U′wais

**Affiliations:** ^1^ Department of Oral and Maxillofacial Surgery, Oral Medicine and Periodontology, School of Dentistry, The University of Jordan, Amman, Jordan, ju.edu.jo; ^2^ Family Medicine, Department of Internal Medicine, Internal Medicine, School of Medicine, The University of Jordan, Amman, Jordan, ju.edu.jo; ^3^ Faculty of Dentistry, Jordan University of Science and Technology, Irbid, Jordan, just.edu.jo

**Keywords:** case report, infraorbital nerve, orbital floor, paresthesia, trauma

## Abstract

Orbital floor fractures are among the most common fractures in the maxillofacial area. Nonetheless, muscular entrapment and enophthalmos are the only known reasons for orbital floor reconstruction, with little data supporting infraorbital nerve paresthesia as an indication. This case report presents a 30‐year‐old female patient who developed chronic infraorbital nerve paresthesia for 3 weeks as a result of an orbital blowout fracture involving the infraorbital canal. The patient underwent infraorbital nerve decompression and orbital floor reconstruction. The outcome was satisfactory, as the patient reported an immediate postoperative neurosensory recovery, confirmed by a light touch test and comparison to the contralateral healthy side, which was confirmed over 6 months of follow‐up. If infraorbital paresthesia lasts more than 2 weeks, orbital floor reconstruction may be considered. However, further research is needed to validate the findings of this report.

## 1. Introduction

The orbit is the bony vault that houses the eyeball, but this vault is very thin in specific areas, especially the floor and the medial wall, which are commonly fractured following blunt trauma to the orbit, resulting in what is known as “the blow‐out” fracture [1, 2].

The established indications for orbital floor reconstructions include muscle entrapment, enophthalmos, and symptomatic diplopia with a positive forced duction test [3]. In addition, orbital blowout fractures commonly occur along the orbital floor, medial to the infraorbital nerve canal. Consequently, the infraorbital nerve may be contused, and the canal deformed, leading to symptomatic paresthesia in the region of its distribution. In many cases, these symptoms resolve spontaneously, and surgical intervention is not required; less commonly, however, the paresthesia may persist and progress to neuropathic pain [3]. Although previous reports have described infraorbital nerve decompression in similar contexts, the available literature remains limited, particularly regarding its use for orbital floor reconstruction [3, 4].

The aim of this report is to present a case of persistent, bothersome infraorbital nerve paresthesia, in which surgery was performed to decompress the infraorbital nerve as a suggested indication for orbital floor reconstruction. This case may contribute to the ongoing discussion about the appropriateness of this intervention for orbital blowout fractures.

## 2. Case Report

A 30‐year‐old female patient was referred from the Family Medicine Department at Jordan University Hospital (JUH) after sustaining facial trauma and complaining of persistent paresthesia over the cheek and the nose for 3 weeks without improvement. She was subsequently referred to the Department of Oral and Maxillofacial Surgery for further evaluation. The patient presented a clear and coherent history of her medical, family, and psychosocial background, including pertinent genetic information. On clinical examination, utilizing light touch testing, no normal sensation was reported over the distribution of the infraorbital nerve; this injury was consistent with severe sensory disturbance likely caused by infraorbital nerve compression. Ocular movements were normal, with a full range of motion in all directions and no signs of movement restriction. Furthermore, there was no enophthalmos, ecchymosis, or subconjunctival hemorrhage. A computed tomography (CT) scan was ordered and revealed a pure blowout fracture of the right orbital floor directly at the site of the infraorbital canal, as well as herniation of the orbital soft tissue into the maxillary sinus (Figures 1 and 2). Three weeks following the trauma, the patient underwent orbital floor exploration and infraorbital nerve decompression under general anesthesia (GA). Tarsorrhaphy was performed using 4‐0 Vicryl to protect the cornea during eye manipulation. The orbital floor was reached via an infraorbital incision (Figure 3). The skin‐muscle flap was elevated, and dissection was performed superfacially to the orbital septum down to the infraorbital rim. A periosteal incision was created right below the infraorbital rim with a needle‐tip cautery, followed by reflection of the periosteum orbital septum to the orbital floor. Decompression of the infraorbital nerve and elevation of the herniated tissues from the maxillary sinus were then performed. The orbital floor defect was covered with a trapezoidal titanium mesh (ANTON‐HIPP), which was secured on the orbital rim with two 4‐mm monocortical screws (Figure 4). A forced duction test was performed and showed a negative result, confirming that globe mobility was intact. The approximation of the facial periosteum to the arcus marginalis was performed with 3‐0 Vicryl suture. A 6‐0 nonresorbable suture (Nylon, Ethicon Johnson, United States) was used in a subcuticular manner, and the wound was covered with a Steri‐Strip. The patient was discharged 1 day after surgery. A postoperative CT scan showed acceptable reduction of the fracture and good adaptation of the titanium mesh (Figures 5 and 6). Overall, the patient tolerated the surgery and was referred to an ophthalmologist, who found normal pupillary responses and retained extraocular movements. The patient was observed for more than 6 months, with no complaint of paresthesia as assessed by light touch and comparison with the contralateral side, and no symptoms of visual disturbances or diplopia. Table 1 summarizes the patient′s historical and most recent details from the episode of care, arranged chronologically.

**Figure 1:**
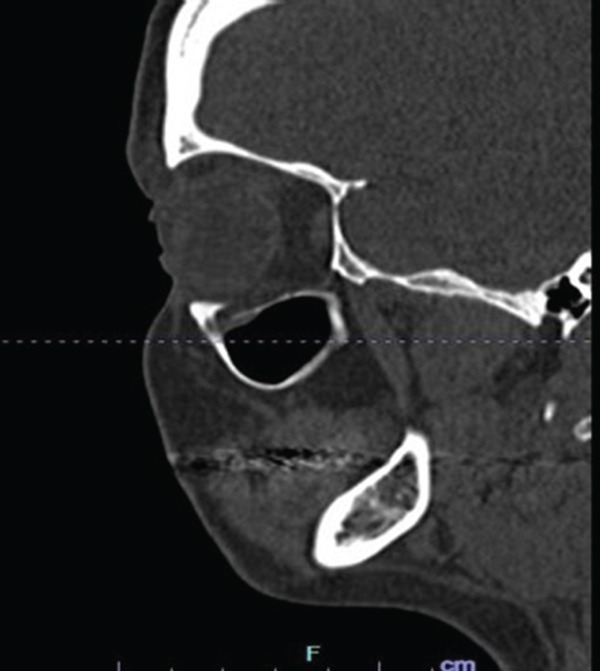
CT scan: Sagittal view showing inferior displacement of orbital floor.

**Figure 2:**
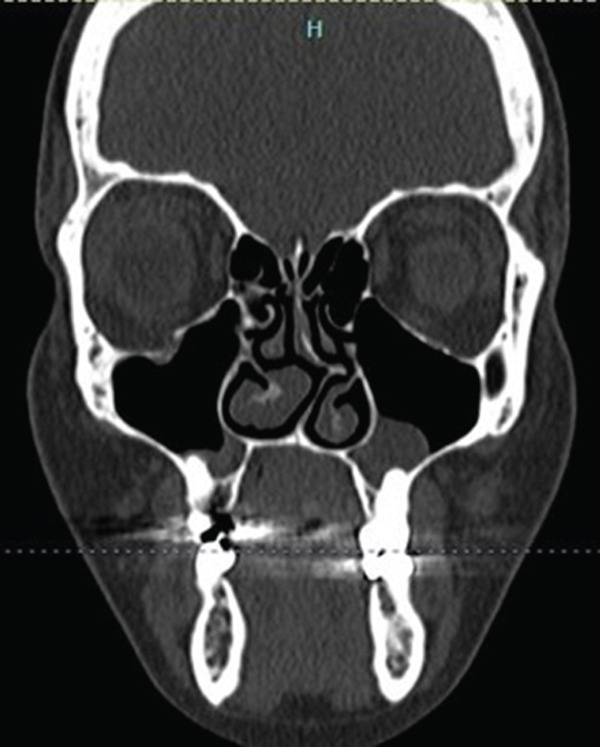
CT scan: Coronal view showing displacement of soft tissue within the maxillary sinus.

**Figure 3:**
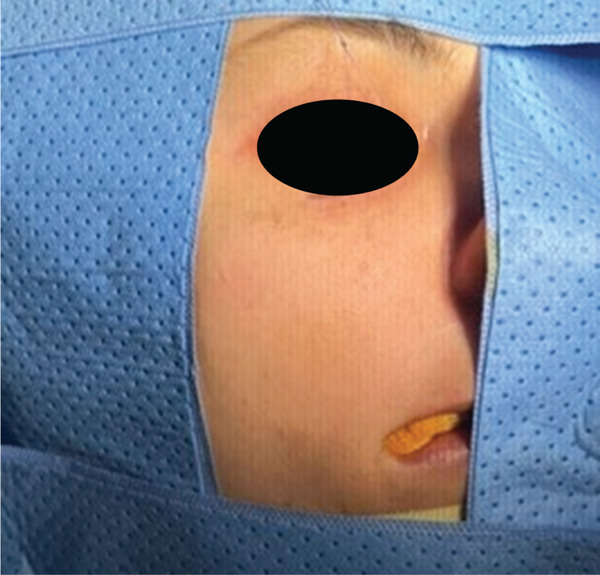
The marking for the infraorbital approach that was used.

**Figure 4:**
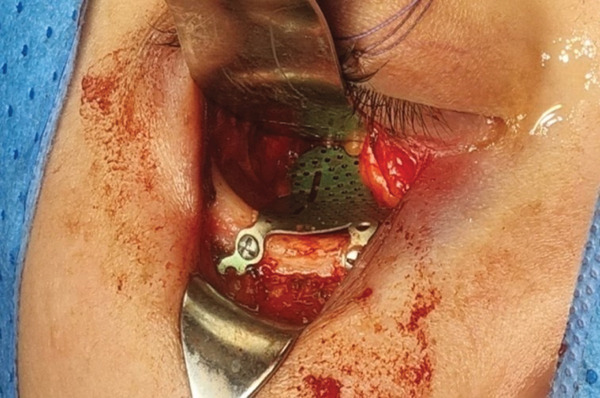
Titanium mesh in place with fixation to the inferior orbital rim.

**Figure 5:**
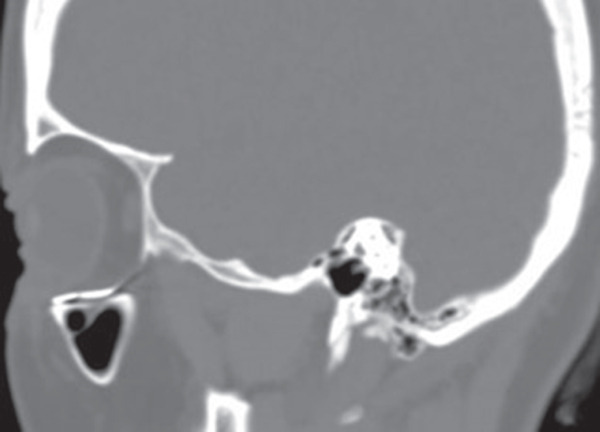
Postoperative sagittal CT showing the posterior extent of the mesh.

**Figure 6:**
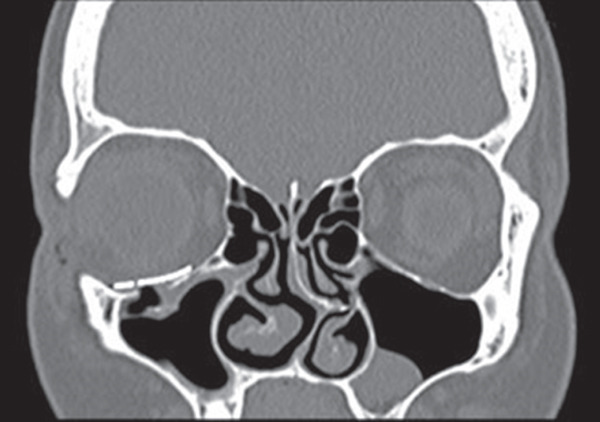
Postoperative CT scan: Coronal view showing good adaptation of the mesh.

**Table 1:** Patient′s historical and most recent details from the episode of care, arranged chronologically.

Timeframe	Event/symptom	Intervention/Outcome
June 2024	Onset of orbital fracture and infraorbital paresthesia	Nothing
July 2024 (after 3 weeks)	First visit to family physician	Referral to the Department of Oral and Maxillofacial Surgery and time elapsed since the trauma was 3 weeks
July 2024 (after 2 days)	Hospital admission for surgery	Orbital floor exploration and reconstruction
January 2025 (after 6 months)	Follow up (Department of Oral and Maxillofacial Surgery)	Examination for paresthesia or any signs of visual disturbances

## 3. Discussion

The infraorbital nerve is located in the floor of the orbit and is anatomically divided into three parts: the foraminal part, which exists through the infraorbital foramen; the canalicular part, which lies within the infraorbital canal; and the infraorbital sulcus part, located after entering the inferior orbital fissure and before entering the infraorbital canal. The foraminal and the canalicular parts are usually subjected to compression‐type trauma, whereas the infraorbital sulcus part is typically affected by shearing forces [4, 5].

Hypoesthesia is defined as abnormally decreased sensitivity to stimulation. Hyperesthesia refers to an abnormal, excessive sensation of pain. Dysesthesia is an unpleasant abnormal sensation produced by normal stimuli. Paresthesia refers to abnormal sensations such as burning, prickling, or formication. Causalgia is a burning pain often accompanied by trophic skin changes due to peripheral nerve injury [4]. Infraorbital nerve injury following orbital trauma is a common finding [6].

The incidence of infraorbital nerve injury has been reported to be 60%–70% in cases of orbital trauma [7]. However, there is limited literature regarding the effect of surgical repair on the healing of infraorbital nerve injury.

The established indications for orbital floor reconstructions include displaced fractures involving more than 50% of the floor, extraocular muscle entrapment, and significant enophthalmos [4]. However, orbital floor reconstruction solely for the treatment of infraorbital injury remains controversial, given the assumption that most cases of paresthesia improve over time. Nevertheless, if traumatic injury to the infraorbital nerve is ignored and not treated promptly, the patient may eventually develop maxillary neuralgia, which is often resistant to both conservative and surgical management [8]. In the present case, the patient was presented with severe, persistent, and infraorbital hypoesthesia. The prognosis was favorable, with a gradual increase in sensation and resolution of paresthesia. Following infraorbital nerve decompression, light touch sensation in the infraorbital nerve distribution was assessed, as previously described in studies investigating infraorbital nerve injuries [9], and the patient′s subjective symptoms improved by more than 90% within 2 months. The favorable prognosis in the present case was based on the presumed mechanism of injury. CT demonstrated deformation of the infraorbital canal at the fracture site, suggesting a compressive rather than a complete transection injury of the infraorbital nerve. Surgical decompression may relieve mechanical pressure on the nerve, restore the local neural environment, and facilitate functional recovery.

It is noteworthy that the necessity of periosteal closure is still debated, especially given the possibility of ectropion or entropion when anatomical layers are improperly sutured [10, 11]. In the present case, the facial periosteum was approximated to the arcus marginalis. This approach was selected intentionally to ensure proper anatomical layering and avoid inappropriate tethering of superficial eyelid structures [11]. Although debate continues, existing research suggests that complications such as early ectropion or lid shortening are more closely related to improper suturing techniques than to periosteal closure [11]. In this case, reapproximating the facial periosteum to the arcus marginalis helped to restore the facial soft tissues to their original position and minimize the risk of postoperative facial sagging, while retaining normal eyelid anatomy.

Although there is limited literature [3, 4] examining the outcomes of orbital floor reconstruction performed solely for infraorbital nerve paresthesia following blowout fractures, one case series reported nine patients with traumatic infraorbital paresthesia who were treated, underwent surgery and experienced significant improvement in their neurological symptoms. Notably, all these patients had minor orbital floor fractures without extraocular muscle entrapment and were initially managed nonoperatively. However, because of persistent neurological symptoms caused by compression of the infraorbital nerve, six of the patients developed depression and reduced quality of life requiring antidepressant therapy due to chronic pain. Surgical decompression of the infraorbital nerve resulted in significant improvement in both neurological symptoms and psychological well‐being in all nine patients [3].

In another case series, two patients underwent surgery for orbital blowout fractures with infraorbital nerve hypoesthesia as the primary indication. The first patient had an orbital floor repair, which included the removal of bone fragments that were compressing the nerve and the insertion of an orbital floor implant. The patient reported an initial recovery of sensation 5 days after surgery, and by 6 months, sensation had returned to 75% of the preinjury levels. The second patient suffered a pure orbital floor blowout fracture with hypoesthesia in the infraorbital nerve distribution area. The patient underwent infraorbital nerve decompression and orbital floor reconstruction. The patient reported partial recovery of sensation beneath the eye 1 week following surgery. The following 3 months, approximately 95% of sensation had recovered, although intermittent pain and paresthesia persisted [4].

Orbital floor fracture reconstruction may be indicated for infraorbital nerve decompression in cases of irreversible anesthesia, for which surgical decompression is recommended within 1–2 weeks after trauma [8]. Furthermore, if irreversible partial disturbance of nerve sensitivity (hypoesthesia) persists for more than 3 weeks, so‐called late‐term decompression is suggested if the hypoesthesia is accompanied by disturbing painful paresthesia or neuralgia. However, cases of reversible infraorbital paresthesia occurring within the first 2 weeks of injury do not require surgical intervention, and a conservative “wait‐and‐see” approach is suggested. Given the current report′s persistent severe infraorbital paresthesia for 3 weeks without improvement, surgical intervention was suggested to relieve possible nerve compression and prevent long‐term neuropathic consequences [8].

Despite the improvement in neurological symptoms reported in the aforementioned literature and observed in the present case, it cannot be confirmed that such improvement is attributable to surgery alone. Therefore, further studies are needed to validate the findings of previous literature. The neurosensory assessment in this case was based on light touch testing, which represents a subjective method. The absence of standardized objective measures, such as two‐point discrimination or Semmes–Weinstein monofilament testing, constitutes a limitation of this report.

The patient was invited to provide her perspective on the treatment she received, reflecting on her experience, perceived effectiveness, and any challenges she encountered. The patient stated that persistent numbness and tingling had a negative impact on her quality of life, including mood and daily activities, but that surgery resulted in significant improvement, with sensation recovered and psychological health improved.

## 4. Conclusion

Infraorbital nerve decompression may represent a potential consideration in selected cases of persistent infraorbital paresthesia following orbital floor fractures. However, within the limitations of the study, these findings should be interpreted with caution, and further well‐designed studies are required to confirm this observation.

## Author Contributions

Conceptualization: **Mohammad H. Al-Shayyab**; methodology: **Mohammad H. Al-Shayyab** and **Ayham Dahooh**; investigation: **Ayham Dahooh** and **Motaz Barakat**; data curation: **Mohammad H. Al-Shayyab** and **Lina Ghattas**; formal analysis: **Mohammad H. Al-Shayyab**; visualization: **Lina Ghattas** and **Sara Abdallah**; writing – original draft preparation: **Ayham Dahooh**, **Mohammad H. Al-Shayyab**, and **Motaz Barakat**; writing – review & editing: **Majd Y. U′wais** and **Lina Ghattas**; supervision: **Mohammad H. Al-Shayyab**.

## Funding

No funding was received for this manuscript.

## Disclosure

All authors have read and approved the final version of the manuscript.

## Ethics Statement

The protocol was reviewed and approved by the Institutional Review Board of the University of Jordan (Approval No. 10/2026/7841), and written informed consent was obtained from the participant.

## Consent

Written informed consent was obtained from the patient for the diagnostic and surgical procedures performed, as well as for the publication of this case report and any accompanying clinical images.

## Conflicts of Interest

The authors declare no conflicts of interest.

## Data Availability

The data that support the findings of this study are available from the corresponding author upon reasonable request.
